# The missing crystal structure in the series of *N*,*N*′,*N*′′-tris­(pyridinyl)benzene-1,3,5-tricarbox­amides: the 2-pyridinyl derivative

**DOI:** 10.1107/S2056989020005599

**Published:** 2020-05-01

**Authors:** Levi Senior, Anthony Linden

**Affiliations:** a Department of Chemistry, University of Zurich, Winterthurerstrasse 190, CH-8057 Zurich, Switzerland

**Keywords:** trimesic amide, benzene­tricarboxamide, hydrogen-bonding, crystal structure

## Abstract

In the first reported crystal structure involving the potential ligand *N*,*N*′,*N*′′-tris­(pyridin-2-­yl)benzene-1,3,5-­tricarboxamide, C_24_H_18_N_6_O_3_, inter­molecular N—H⋯O hydrogen bonds link the mol­ecules *via* their amide groups into slanted ladder-like chains, in which the uprights of the ladder are formed by the hydrogen-bonding inter­actions and the benzene ring cores of the mol­ecules act as the rungs of the ladder. Only two of the three amide groups in the mol­ecule are involved in hydrogen bonding and this influences the degree of out-of-plane twisting at each amide group, with the twist being more significant for those amide groups participating in hydrogen bonds.

## Abstract 2

In the first reported crystal structure involving the potential ligand *N*,*N*′,*N*′′-tris­(2-pyridin­yl)-1,3,5-benzene­tricarboxamide, inter­molecular N—H⋯O hydrogen bonds link the mol­ecules *via* their amide groups into slanted ladder-like chains. Only two of the three amide groups in the mol­ecule are involved in hydrogen bonding, which influences the degree of out-of-plane twisting at each amide group.

## Chemical context   

Branched coordinating ligands with potential donor atoms on each branch can be useful as spacers in the synthesis of coordination polymers and metal–organic frameworks. A frequently used starting material is benzene-1,3,5-tri­carb­oxy­lic acid (trimesic acid), which can act as a three-way planar node-connector. A related, less frequently employed, ligand system is *N*,*N*′,*N*′′-tris­(*n*-pyridin­yl)-1,3,5-benzene­tricarboxamide (*n* = 2, 3 or 4), which has potential donor atoms on each pyridinyl ring and at the amide function.
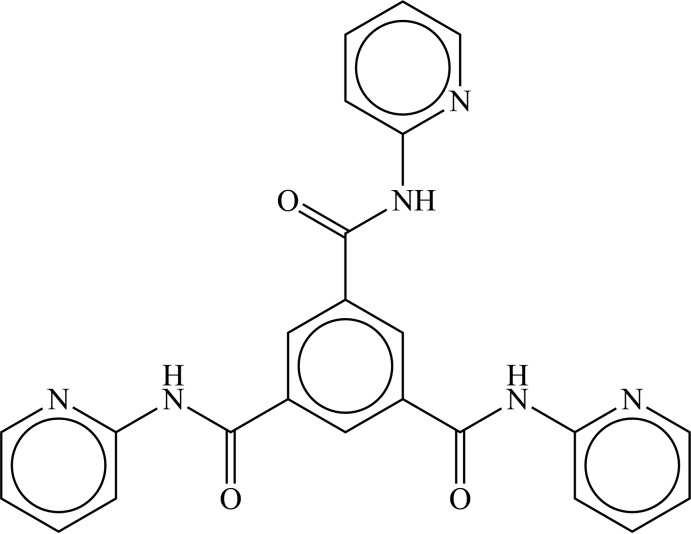


We are inter­ested in constructing bis­muth(III) coordination polymers (Senior & Linden, 2020*a* ▸,*b* ▸) and have synthesized the above three amides as potential spacer ligands, although, so far, experiments involving these have not produced any Bi^III^ coordination polymers. The crystal structure of the 2-pyridinyl derivative, *N*,*N*′,*N*′′-tris­(2-pyridin­yl)-1,3,5-benzene­tricarboxamide, C_24_H_18_N_6_O_3_, (I), has not previously been reported and is described here.

## Structural commentary   

**Figure 1:**
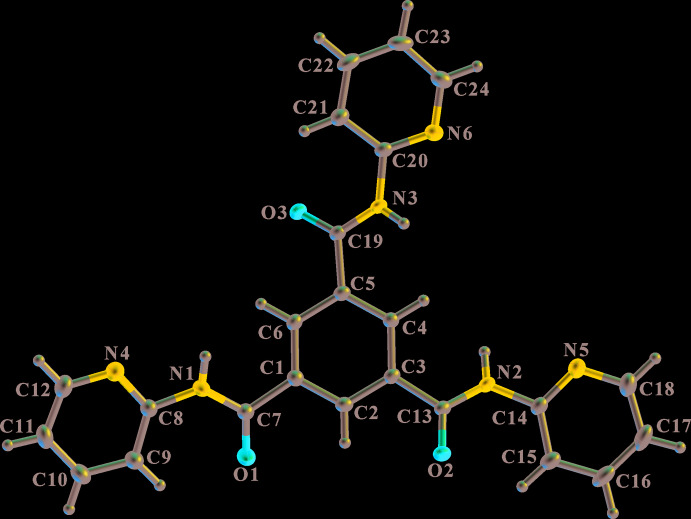
View of the asymmetric unit of (I) showing the atom-labelling scheme. Displacement ellipsoids are drawn at the 50% probability level. H atoms are represented by circles of arbitrary size.

The asymmetric unit of (I), shown in Fig. 1 ▸, contains one mol­ecule, which, despite its chemical threefold symmetry, does not adopt any crystallographic symmetry, nor does it have a propeller-like conformation in which the orientations of the amide groups all lie with the same relative orientation as one progresses around the benzene ring. This may be related to the absence of any hydrogen-bonding inter­actions at one of the amide groups, while the other amide groups act as both hydrogen-bond donors and acceptors (see *Supra­molecular features*). The 2-pyridinyl rings all lie with the ring N atom approximately *cis* to the amide N—H group.

The bond lengths and angles in the mol­ecule have normal values and the bond lengths around the amide groups (Table 1 ▸) are not significantly influenced by the presence or absence of hydrogen-bonding inter­actions. Of more inter­est are the deviations from the central benzene ring plane of the amide and 2-pyridinyl groups. The torsion angles listed in Table 1 ▸ indicate that each of the amide C(O)—N bonds is twisted by approximately 27° out of the plane of the benzene ring. On the other hand, for the amide group not involved in hydrogen bonding, the amide C7(O1)—N1 bond is twisted by less than 8° from the plane of the 2-pyridinyl ring, while for the other two amide groups, the magnitude of the C(O)—N twist is in the range 25–34°. This suggests that the hydrogen-bonding inter­actions significantly influence the orientation of the adjacent 2-pyridinyl ring; where inter­actions occur, the ring is rotated more to accommodate the inter­molecular hydrogen bonds. The dihedral angles between the planes of the benzene ring and the 2-pyridinyl rings adjacent to the amide groups involving atoms N1, N3 and N5 are 20.41 (5), 3.11 (5) and 7.50 (5)°, respectively, which again highlights the difference attributable to the absence of hydrogen-bonding inter­actions at the amide group involving atom N1.

## Supra­molecular features   

**Figure 2:**
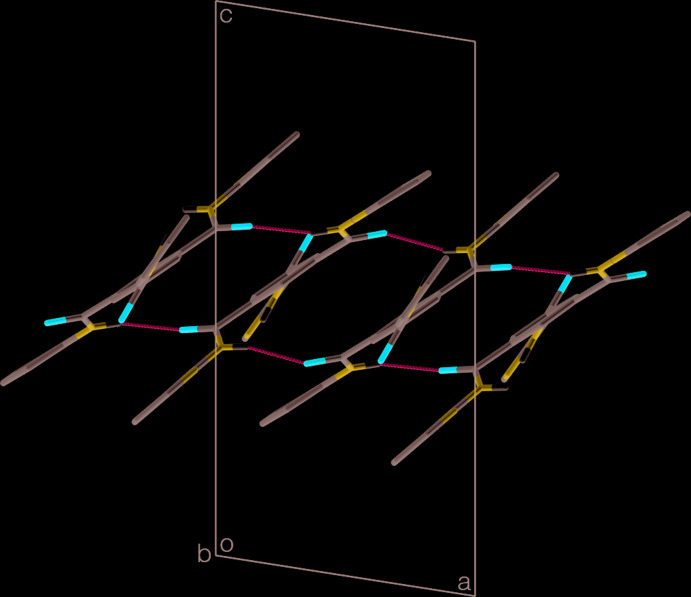
One of the hydrogen-bonded supra­molecular ladders in (I) viewed down the *b* axis. H atoms bonded to C atoms have been omitted for clarity.

In the extended structure of (I), the mol­ecules are linked into slanted ladder-like chains by N—H⋯O hydrogen bonds, which involve the amide groups as donors and acceptors (Table 2 ▸, Fig. 2 ▸). The 2-pyridinyl N atoms are not involved in these inter­actions. The ladders progress parallel to the [100] direction. The uprights of the ladder are formed by the hydrogen-bonding inter­actions and the benzene ring cores of the mol­ecules act as the rungs of the ladder. Considered separately, amide group N2—H inter­acts with the O atom of the N3—H amide group of an adjacent mol­ecule and continuing the same path brings one back to the original mol­ecule, thereby completing a loop that can be described by a graph-set motif (Bernstein *et al.*, 1995 ▸) of 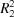
(16). Similarly, the amide group N3—H inter­acts with the O atom of the N2—H amide group of the adjacent mol­ecule on the other side to give the same loop motif. These two loops alternate as one progresses along the ladder. The rungs of the ladder can be described by the chain graph-set motif of 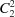
(8), because it involves the N2—H and N3—H amide groups in an alternating sequence.

The slanted stacking of the mol­ecules as the rungs of the hydrogen-bonded ladder only allow weak π–π inter­actions, which occur between the central benzene ring and the 2-pyridinyl ring containing atom N5 in the centrosym­metrically related adjacent mol­ecule on one side at 1 − *x*, 1 − *y*, 1 − *z*, and with the 2-pyridinyl ring containing atom N6 in the centrosymmetrically related adjacent mol­ecule on the other side at −*x*, 1 − *y*, 1 − *z*. These inter­actions reinforce the ladder structure, rather than linking adjacent ladders. For the inter­actions involving the 2-pyridinyl rings containing atoms N5 and N6, respectively, the distances between the centroids of the benzene and 2-pyridinyl rings are 3.8956 (6) and 3.8409 (6) Å, the perpendicular distances between the centroid of the benzene ring and the planes of the 2-pyridinyl rings are 3.4522 (5) and 3.4610 (4) Å, while the slippages of the centroids are 1.735 and 2.097 Å and the angles between the benzene and 2-pyridinyl ring planes are 3.11 (5) and 7.50 (5)°.

## Database survey   

The Cambridge Structural Database (CSD, version 5.41, update of March 2020; Groom *et al.*, 2016 ▸) has no entries for (I), its salts, nor for its use as a ligand. There are six crystal structures reported for the tris­(3-pyridin­yl) analogue; the pure ansolvate (Palmans *et al.*, 1997 ▸), and five reports of the monohydrate, which occurs in two polymorphic forms with space groups *Cc* and *Pbca* (Rajput & Biradha, 2008 ▸, 2011 ▸; Jia *et al.*, 2009 ▸; Zhang *et al.*, 2016 ▸). In the ansolvate, the 3-pyridinyl rings all lie with the ring N atom approximately *cis* to the adjacent amide N—H group, as in (I), but is the only example among the 2- and 3-pyridinyl analogues where a propellor-like sequence of the three arms of the mol­ecule is observed. In the *Cc* polymorph of the monohydrate, two of the 3-pyridinyl rings lie approximately *trans* to their adjacent amide N—H groups, while in the *Pbca* polymorph, all three of the 3-pyridinyl rings have the *trans* arrangement. Surprisingly, there are only three crystal structures reported for the tris­(4-pyridin­yl) analogue; the monohydrate (Rajput & Biradha, 2011 ▸), its chloro­form solvate monohydrate (Luo *et al.*, 2013 ▸) and its di­methyl­sulfoxide methanol solvate (Kumar *et al.*, 2004 ▸). Only the latter two display a propeller-like sequence of the three arms of the mol­ecule

The CSD contains 28 entries for coordination complexes where the tris­(3-pridin­yl) analogue acts as a ligand. In most of these, the ligand coordinates through the pyridinyl N atom, although the amide O atom is involved in a few examples. The tris­(4-pyridin­yl) analogue occurs as a ligand in six coordination complexes, all of which involve coordination through the pyridinyl N atom. Given the propensity of the pyridinyl N atom to act as the coordinating atom in these examples, the steric congestion between the 2-pyridinyl ring and the adjacent amide group of (I) might indicate why it has not appeared as a ligand in any coordination complexes so far. Presumably for similar reasons, the CSD does not contain entries involving analogous mol­ecules or ligands where the 2-pyridinyl rings have been replaced by 2-benzoic acid or 2-benzoate substituents and there are no known reports of their synthesis. The CSD contains entries for seven and 19 complexes with the 3-and 4-benzoate ligands, respectively, but only one crystal structure involving a neutral acid, that of the tris­(4-benzoic acid) analogue, is known (Zhang *et al.*, 2012 ▸).

## Synthesis and crystallization   

A solution of 2-amino­pyridine (0.96 g) in di­chloro­methane (DCM) (12 ml) and tri­methyl­amine (TEA) (1.4 ml) was added dropwise to a solution of benzene-1,3,5-tri­carb­oxy­lic acid trichloride in DCM (3.4 ml) at 273 K. A further 1.5 ml of TEA were added and reaction mixture stirred at room temperature for approximately 5 days until the dark-brown–red slurry turned yellow–orange. The reaction mixture was filtered under vacuum and washed with DCM. It proved difficult to isolate a purified product until the product was washed with copious amounts of water, then collected as a precipitate *via* filtration through fluted filter paper and dried in air between filter papers. Crystals were grown by dissolving the product in warm methanol, filtering and allowing slow evaporation of solvent overnight. A small qu­antity of orange–yellow crystals was recovered from a yellow oil. ^1^H NMR δ: 11.03 (*s*, 3H), 8.76 (*s*, 3H), 8.44 (*d*, 3H, *J* = 4.82), 8.28 (*d*, 3H, *J* = 8.48), 7.90 (*t*, 3H, *J* = 7.90), 7.22 (*t*, 3H, *J* = 6.16); ESI *m*/*z*: 438.14335 (predicted 438.14).

## Refinement   

Crystal data, data collection and structure refinement details are summarized in Table 3 ▸. The amide H atoms were located in a difference-Fourier map and their positions were refined together with individual isotropic displacement parameters. All other H atoms were placed in geometrically idealized positions and constrained to ride on their parent atoms (C—H = 0.95 Å) with *U*_iso_(H) = 1.2*U*_eq_(C).

## Supplementary Material

Crystal structure: contains datablock(s) I, global. DOI: 10.1107/S2056989020005599/hb7909sup1.cif

Structure factors: contains datablock(s) I. DOI: 10.1107/S2056989020005599/hb7909Isup2.hkl

Supporting information file. DOI: 10.1107/S2056989020005599/hb7909Isup3.cml

CCDC reference: 1998283

Additional supporting information:  crystallographic information; 3D view; checkCIF report

## Figures and Tables

**Table 1:** Selected geometric parameters (Å, °)

N1—C7	1.3578 (14)	N2—C14	1.4163 (12)
N1—C8	1.4085 (14)	N3—C19	1.3468 (13)
N2—C13	1.3499 (13)	N3—C20	1.4195 (12)
			
N1—C7—C1—C6	−28.16 (15)	C13—N2—C14—C15	25.72 (15)
C7—N1—C8—C9	7.5 (2)	N3—C19—C5—C4	−27.77 (13)
N2—C13—C3—C4	−26.51 (13)	C19—N3—C20—C21	33.81 (15)

**Table 2:** Hydrogen-bond geometry (Å, °)

*D*—H⋯*A*	*D*—H	H⋯*A*	*D*⋯*A*	*D*—H⋯*A*
N2—H2⋯O3^i^	0.898 (14)	2.108 (14)	2.9866 (11)	165.6 (13)
N3—H3⋯O2^ii^	0.857 (14)	2.054 (14)	2.8781 (11)	160.9 (13)

Symmetry codes: (i) 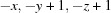; (ii) 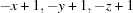.

**Table 3:** Experimental details

Crystal data	
Chemical formula	C_24_H_18_N_6_O_3_
*M*_r_	438.44
Crystal system, space group	Monoclinic, *P*2_1_/*n*
Temperature (K)	160
*a*, *b*, *c* (Å)	8.2807 (1), 14.1554 (1), 17.5020 (2)
β (°)	98.920 (1)
*V* (Å^3^)	2026.71 (4)
*Z*	4
Radiation type	Cu *K*α
μ (mm^−1^)	0.81
Crystal size (mm)	0.24 × 0.09 × 0.09

Data collection	
Diffractometer	Oxford Diffraction SuperNova, dual radiation diffractometer
Absorption correction	Multi-scan (*CrysAlis PRO*; Rigaku OD, 2017 ▸)
*T*_min_, *T*_max_	0.898, 1.000
No. of measured, independent and observed [*I* > 2σ(*I*)] reflections	19285, 4020, 3786
*R*_int_	0.018
(sin θ/λ)_max_ (Å^−1^)	0.624

Refinement	
*R*[*F* ^2^ > 2σ(*F* ^2^)], *wR*(*F* ^2^), *S*	0.031, 0.088, 1.05
No. of reflections	4020
No. of parameters	311
H-atom treatment	H atoms treated by a mixture of independent and constrained refinement
Δρ_max_, Δρ_min_ (e Å^−3^)	0.27, −0.17

Computer programs: *CrysAlis PRO* (Rigaku OD, 2017 ▸), *SHELXT2018* (Sheldrick, 2015*a* ▸), *SHELXL2018* (Sheldrick, 2015*b* ▸), *OLEX2* (Dolomanov *et al.*, 2009 ▸), *Mercury* (Macrae *et al.*, 2020 ▸) and *PLATON* (Spek, 2015 ▸, 2020 ▸).

## supplementary crystallographic information

### Crystal data

**Table:**

C_24_H_18_N_6_O_3_	*F*(000) = 912
*M**_r_* = 438.44	*D*_x_ = 1.437 Mg m^−^^3^
Monoclinic, *P*2_1_/*n*	Cu *K*α radiation, λ = 1.54184 Å
*a* = 8.2807 (1) Å	Cell parameters from 12896 reflections
*b* = 14.1554 (1) Å	θ = 3.1–74.1°
*c* = 17.5020 (2) Å	µ = 0.81 mm^−^^1^
β = 98.920 (1)°	*T* = 160 K
*V* = 2026.71 (4) Å^3^	Prism, pale yellow
*Z* = 4	0.24 × 0.09 × 0.09 mm

### Data collection

**Table:**

Oxford Diffraction SuperNova, dual radiation diffractometer	4020 independent reflections
Radiation source: micro-focus sealed X-ray tube, SuperNova (Cu) X-ray source	3786 reflections with *I* > 2σ(*I*)
Mirror monochromator	*R*_int_ = 0.018
Detector resolution: 10.3801 pixels mm^-1^	θ_max_ = 74.2°, θ_min_ = 4.0°
ω scans	*h* = −10→10
Absorption correction: multi-scan (CrysAlisPro; Rigaku OD, 2017)	*k* = −17→17
*T*_min_ = 0.898, *T*_max_ = 1.000	*l* = −21→18
19285 measured reflections	

### Refinement

**Table:**

Refinement on *F*^2^	Secondary atom site location: difference Fourier map
Least-squares matrix: full	Hydrogen site location: difference Fourier map
*R*[*F*^2^ > 2σ(*F*^2^)] = 0.031	H atoms treated by a mixture of independent and constrained refinement
*wR*(*F*^2^) = 0.088	*w* = 1/[σ^2^(*F*_o_^2^) + (0.0474*P*)^2^ + 0.5699*P*] where *P* = (*F*_o_^2^ + 2*F*_c_^2^)/3
*S* = 1.05	(Δ/σ)_max_ = 0.001
4020 reflections	Δρ_max_ = 0.27 e Å^−^^3^
311 parameters	Δρ_min_ = −0.17 e Å^−^^3^
0 restraints	Extinction correction: SHELXL-2018 (Sheldrick, 2015b), Fc^*^=kFc[1+0.001xFc^2^λ^3^/sin(2θ)]^-1/4^
Primary atom site location: dual	Extinction coefficient: 0.0012 (2)

### Special details

**Table:**

Experimental. Data collection and full structure determination done by Prof. Anthony Linden: anthony.linden@chem.uzh.chSolvent used: methanol Cooling Device: Oxford Instruments Cryojet XL Crystal mount: on a glass fibre Frames collected: 1718 Seconds exposure per frame: 1.0-5.0 Degrees rotation per frame: 1.0 Crystal-detector distance (mm): 55.0 Client: Levi Senior Sample code: LS002
Geometry. All esds (except the esd in the dihedral angle between two l.s. planes) are estimated using the full covariance matrix. The cell esds are taken into account individually in the estimation of esds in distances, angles and torsion angles; correlations between esds in cell parameters are only used when they are defined by crystal symmetry. An approximate (isotropic) treatment of cell esds is used for estimating esds involving l.s. planes.

### Fractional atomic coordinates and isotropic or equivalent isotropic displacement parameters (Å^2^)

**Table:**

	*x*	*y*	*z*	*U*_iso_*/*U*_eq_	
O1	0.36342 (11)	0.88417 (6)	0.60262 (5)	0.0380 (2)	
O2	0.64957 (8)	0.57226 (5)	0.62868 (4)	0.02469 (18)	
O3	−0.13032 (8)	0.61942 (5)	0.39693 (4)	0.02547 (18)	
N1	0.22689 (12)	0.90845 (6)	0.48097 (6)	0.0286 (2)	
H1	0.1826 (19)	0.8797 (11)	0.4388 (9)	0.044 (4)*	
N2	0.47719 (10)	0.44612 (6)	0.62364 (5)	0.02113 (19)	
H2	0.3752 (17)	0.4245 (10)	0.6088 (8)	0.034 (4)*	
N3	0.02167 (10)	0.49006 (6)	0.37699 (5)	0.02058 (19)	
H3	0.1165 (18)	0.4642 (10)	0.3849 (8)	0.033 (4)*	
N4	0.12823 (13)	1.03623 (7)	0.41011 (6)	0.0330 (2)	
N5	0.53774 (11)	0.28992 (6)	0.64545 (6)	0.0271 (2)	
N6	−0.07968 (11)	0.34722 (6)	0.32839 (5)	0.0249 (2)	
C1	0.27688 (12)	0.74907 (7)	0.52677 (6)	0.0211 (2)	
C2	0.39380 (12)	0.69079 (7)	0.56837 (6)	0.0208 (2)	
H201	0.481926	0.717841	0.602596	0.025*	
C3	0.38289 (11)	0.59313 (7)	0.56032 (5)	0.0185 (2)	
C4	0.25660 (11)	0.55341 (7)	0.50819 (6)	0.0183 (2)	
H4	0.249923	0.486807	0.501836	0.022*	
C5	0.14014 (11)	0.61163 (7)	0.46542 (6)	0.0187 (2)	
C6	0.14907 (12)	0.70904 (7)	0.47598 (6)	0.0205 (2)	
H6	0.067381	0.748521	0.448346	0.025*	
C7	0.29370 (12)	0.85343 (7)	0.54121 (6)	0.0245 (2)	
C8	0.21855 (13)	1.00767 (7)	0.47569 (6)	0.0250 (2)	
C9	0.29662 (15)	1.06853 (8)	0.53146 (7)	0.0338 (3)	
H9	0.360791	1.045229	0.577297	0.041*	
C10	0.27774 (16)	1.16473 (8)	0.51797 (8)	0.0375 (3)	
H10	0.329125	1.208782	0.554908	0.045*	
C11	0.18406 (15)	1.19619 (8)	0.45073 (7)	0.0335 (3)	
H11	0.169393	1.261778	0.440413	0.040*	
C12	0.11257 (15)	1.12956 (8)	0.39909 (7)	0.0351 (3)	
H12	0.048082	1.151096	0.352713	0.042*	
C13	0.51468 (11)	0.53603 (7)	0.60758 (6)	0.0189 (2)	
C14	0.58508 (12)	0.37816 (7)	0.66292 (6)	0.0213 (2)	
C15	0.72325 (13)	0.40119 (8)	0.71560 (6)	0.0278 (2)	
H15	0.750539	0.465105	0.728164	0.033*	
C16	0.81959 (14)	0.32752 (9)	0.74906 (7)	0.0354 (3)	
H16	0.916222	0.340277	0.784429	0.043*	
C17	0.77409 (14)	0.23553 (9)	0.73062 (7)	0.0367 (3)	
H17	0.839025	0.184035	0.752419	0.044*	
C18	0.63199 (14)	0.22034 (8)	0.67976 (7)	0.0331 (3)	
H18	0.599003	0.156956	0.668379	0.040*	
C19	−0.00171 (11)	0.57413 (7)	0.40961 (6)	0.0194 (2)	
C20	−0.09362 (11)	0.44064 (7)	0.32296 (6)	0.0201 (2)	
C21	−0.20550 (13)	0.48708 (8)	0.26842 (6)	0.0284 (2)	
H21	−0.208759	0.554101	0.265813	0.034*	
C22	−0.31225 (14)	0.43239 (9)	0.21791 (7)	0.0352 (3)	
H22	−0.391456	0.461449	0.180111	0.042*	
C23	−0.30220 (14)	0.33527 (9)	0.22314 (7)	0.0338 (3)	
H23	−0.374646	0.296219	0.189504	0.041*	
C24	−0.18422 (14)	0.29632 (8)	0.27848 (7)	0.0301 (2)	
H24	−0.176591	0.229419	0.281387	0.036*	

### Atomic displacement parameters (Å^2^)

**Table:**

	*U*^11^	*U*^22^	*U*^33^	*U*^12^	*U*^13^	*U*^23^
O1	0.0526 (5)	0.0223 (4)	0.0315 (5)	0.0017 (3)	−0.0178 (4)	−0.0036 (3)
O2	0.0165 (3)	0.0221 (4)	0.0330 (4)	0.0004 (3)	−0.0039 (3)	−0.0040 (3)
O3	0.0167 (3)	0.0225 (4)	0.0345 (4)	0.0024 (3)	−0.0045 (3)	−0.0016 (3)
N1	0.0375 (5)	0.0185 (4)	0.0254 (5)	−0.0017 (4)	−0.0088 (4)	−0.0016 (4)
N2	0.0151 (4)	0.0218 (4)	0.0246 (4)	0.0011 (3)	−0.0029 (3)	0.0014 (3)
N3	0.0142 (4)	0.0212 (4)	0.0243 (4)	0.0006 (3)	−0.0033 (3)	−0.0009 (3)
N4	0.0390 (5)	0.0252 (5)	0.0306 (5)	0.0004 (4)	−0.0080 (4)	0.0027 (4)
N5	0.0246 (4)	0.0237 (5)	0.0316 (5)	0.0024 (3)	0.0002 (4)	0.0032 (4)
N6	0.0235 (4)	0.0226 (4)	0.0269 (5)	−0.0015 (3)	−0.0011 (3)	−0.0019 (3)
C1	0.0214 (5)	0.0191 (5)	0.0220 (5)	−0.0002 (4)	0.0010 (4)	−0.0009 (4)
C2	0.0187 (4)	0.0217 (5)	0.0211 (5)	−0.0012 (4)	−0.0002 (4)	−0.0021 (4)
C3	0.0157 (4)	0.0208 (5)	0.0184 (5)	0.0016 (4)	0.0011 (3)	−0.0003 (4)
C4	0.0167 (4)	0.0181 (4)	0.0198 (5)	−0.0003 (3)	0.0017 (4)	−0.0006 (4)
C5	0.0152 (4)	0.0209 (5)	0.0194 (5)	−0.0008 (3)	0.0007 (4)	0.0002 (4)
C6	0.0187 (4)	0.0200 (5)	0.0217 (5)	0.0020 (4)	−0.0002 (4)	0.0016 (4)
C7	0.0250 (5)	0.0203 (5)	0.0260 (5)	0.0008 (4)	−0.0033 (4)	−0.0013 (4)
C8	0.0264 (5)	0.0199 (5)	0.0271 (5)	−0.0004 (4)	−0.0010 (4)	0.0007 (4)
C9	0.0409 (6)	0.0229 (5)	0.0330 (6)	−0.0010 (5)	−0.0090 (5)	−0.0006 (4)
C10	0.0456 (7)	0.0224 (6)	0.0411 (7)	−0.0032 (5)	−0.0042 (5)	−0.0048 (5)
C11	0.0385 (6)	0.0201 (5)	0.0419 (7)	0.0033 (4)	0.0064 (5)	0.0044 (5)
C12	0.0399 (6)	0.0283 (6)	0.0344 (6)	0.0050 (5)	−0.0032 (5)	0.0074 (5)
C13	0.0166 (4)	0.0206 (5)	0.0185 (5)	0.0022 (3)	0.0000 (3)	−0.0039 (4)
C14	0.0181 (5)	0.0244 (5)	0.0209 (5)	0.0025 (4)	0.0020 (4)	0.0033 (4)
C15	0.0242 (5)	0.0328 (6)	0.0241 (5)	−0.0008 (4)	−0.0033 (4)	0.0053 (4)
C16	0.0256 (5)	0.0469 (7)	0.0305 (6)	0.0014 (5)	−0.0057 (4)	0.0143 (5)
C17	0.0303 (6)	0.0399 (7)	0.0389 (7)	0.0108 (5)	0.0025 (5)	0.0185 (5)
C18	0.0327 (6)	0.0260 (6)	0.0402 (7)	0.0056 (5)	0.0042 (5)	0.0082 (5)
C19	0.0162 (4)	0.0198 (5)	0.0210 (5)	−0.0014 (3)	−0.0006 (4)	0.0031 (4)
C20	0.0159 (4)	0.0234 (5)	0.0203 (5)	−0.0013 (4)	0.0007 (4)	−0.0011 (4)
C21	0.0283 (5)	0.0277 (5)	0.0262 (6)	0.0026 (4)	−0.0049 (4)	0.0005 (4)
C22	0.0298 (6)	0.0438 (7)	0.0272 (6)	0.0041 (5)	−0.0103 (5)	−0.0030 (5)
C23	0.0266 (5)	0.0411 (7)	0.0310 (6)	−0.0065 (5)	−0.0039 (4)	−0.0110 (5)
C24	0.0296 (5)	0.0261 (5)	0.0332 (6)	−0.0054 (4)	0.0005 (4)	−0.0064 (4)

### Geometric parameters (Å, º)

**Table:**

O1—C7	1.2189 (13)	C5—C6	1.3916 (13)
O2—C13	1.2325 (12)	C5—C19	1.5032 (13)
O3—C19	1.2334 (12)	C6—H6	0.9500
N1—C7	1.3578 (14)	C8—C9	1.3846 (15)
N1—C8	1.4085 (14)	C9—C10	1.3867 (16)
N1—H1	0.872 (16)	C9—H9	0.9500
N2—C13	1.3499 (13)	C10—C11	1.3792 (18)
N2—C14	1.4163 (12)	C10—H10	0.9500
N2—H2	0.898 (14)	C11—C12	1.3751 (17)
N3—C19	1.3468 (13)	C11—H11	0.9500
N3—C20	1.4195 (12)	C12—H12	0.9500
N3—H3	0.857 (14)	C14—C15	1.3923 (14)
N4—C8	1.3319 (14)	C15—C16	1.3864 (16)
N4—C12	1.3384 (15)	C15—H15	0.9500
N5—C14	1.3304 (14)	C16—C17	1.3798 (19)
N5—C18	1.3395 (14)	C16—H16	0.9500
N6—C20	1.3295 (14)	C17—C18	1.3778 (18)
N6—C24	1.3406 (14)	C17—H17	0.9500
C1—C2	1.3890 (14)	C18—H18	0.9500
C1—C6	1.3927 (13)	C20—C21	1.3883 (14)
C1—C7	1.5015 (14)	C21—C22	1.3855 (16)
C2—C3	1.3910 (14)	C21—H21	0.9500
C2—H201	0.9500	C22—C23	1.3795 (18)
C3—C4	1.3951 (13)	C22—H22	0.9500
C3—C13	1.4996 (13)	C23—C24	1.3795 (16)
C4—C5	1.3949 (13)	C23—H23	0.9500
C4—H4	0.9500	C24—H24	0.9500
			
C7—N1—C8	129.27 (9)	C12—C11—C10	117.85 (11)
C7—N1—H1	117.2 (10)	C12—C11—H11	121.1
C8—N1—H1	113.5 (10)	C10—C11—H11	121.1
C13—N2—C14	126.40 (8)	N4—C12—C11	124.06 (11)
C13—N2—H2	119.8 (9)	N4—C12—H12	118.0
C14—N2—H2	113.8 (9)	C11—C12—H12	118.0
C19—N3—C20	126.29 (8)	O2—C13—N2	123.84 (9)
C19—N3—H3	119.8 (9)	O2—C13—C3	119.29 (9)
C20—N3—H3	113.7 (9)	N2—C13—C3	116.86 (8)
C8—N4—C12	116.92 (10)	N5—C14—C15	123.69 (10)
C14—N5—C18	117.19 (10)	N5—C14—N2	112.64 (9)
C20—N6—C24	116.60 (9)	C15—C14—N2	123.65 (10)
C2—C1—C6	119.46 (9)	C16—C15—C14	117.61 (11)
C2—C1—C7	117.21 (9)	C16—C15—H15	121.2
C6—C1—C7	123.31 (9)	C14—C15—H15	121.2
C1—C2—C3	120.54 (9)	C17—C16—C15	119.52 (11)
C1—C2—H201	119.7	C17—C16—H16	120.2
C3—C2—H201	119.7	C15—C16—H16	120.2
C2—C3—C4	119.81 (9)	C18—C17—C16	118.25 (11)
C2—C3—C13	116.71 (8)	C18—C17—H17	120.9
C4—C3—C13	123.44 (9)	C16—C17—H17	120.9
C5—C4—C3	119.91 (9)	N5—C18—C17	123.69 (11)
C5—C4—H4	120.0	N5—C18—H18	118.2
C3—C4—H4	120.0	C17—C18—H18	118.2
C6—C5—C4	119.73 (9)	O3—C19—N3	124.04 (9)
C6—C5—C19	117.15 (8)	O3—C19—C5	119.95 (9)
C4—C5—C19	123.07 (9)	N3—C19—C5	116.00 (8)
C5—C6—C1	120.49 (9)	N6—C20—C21	124.15 (9)
C5—C6—H6	119.8	N6—C20—N3	113.61 (9)
C1—C6—H6	119.8	C21—C20—N3	122.20 (9)
O1—C7—N1	124.07 (10)	C22—C21—C20	117.76 (11)
O1—C7—C1	121.24 (9)	C22—C21—H21	121.1
N1—C7—C1	114.69 (9)	C20—C21—H21	121.1
N4—C8—C9	123.85 (10)	C23—C22—C21	119.24 (11)
N4—C8—N1	111.99 (9)	C23—C22—H22	120.4
C9—C8—N1	124.15 (10)	C21—C22—H22	120.4
C8—C9—C10	117.58 (11)	C24—C23—C22	118.28 (10)
C8—C9—H9	121.2	C24—C23—H23	120.9
C10—C9—H9	121.2	C22—C23—H23	120.9
C11—C10—C9	119.74 (11)	N6—C24—C23	123.93 (11)
C11—C10—H10	120.1	N6—C24—H24	118.0
C9—C10—H10	120.1	C23—C24—H24	118.0
			
C6—C1—C2—C3	−0.78 (15)	C2—C3—C13—O2	−25.12 (13)
C7—C1—C2—C3	177.55 (9)	C4—C3—C13—O2	152.69 (9)
C1—C2—C3—C4	2.12 (14)	C2—C3—C13—N2	155.68 (9)
C1—C2—C3—C13	−179.99 (9)	N2—C13—C3—C4	−26.51 (13)
C2—C3—C4—C5	−1.17 (14)	C18—N5—C14—C15	−1.58 (16)
C13—C3—C4—C5	−178.92 (9)	C18—N5—C14—N2	−179.99 (9)
C3—C4—C5—C6	−1.09 (14)	C13—N2—C14—N5	−155.86 (10)
C3—C4—C5—C19	−178.46 (9)	C13—N2—C14—C15	25.72 (15)
C4—C5—C6—C1	2.44 (14)	N5—C14—C15—C16	2.67 (16)
C19—C5—C6—C1	179.97 (9)	N2—C14—C15—C16	−179.09 (10)
C2—C1—C6—C5	−1.51 (15)	C14—C15—C16—C17	−1.39 (17)
C7—C1—C6—C5	−179.73 (9)	C15—C16—C17—C18	−0.75 (18)
C8—N1—C7—O1	−1.7 (2)	C14—N5—C18—C17	−0.81 (17)
C8—N1—C7—C1	178.72 (10)	C16—C17—C18—N5	1.96 (19)
C2—C1—C7—O1	−25.98 (15)	C20—N3—C19—O3	0.87 (16)
C6—C1—C7—O1	152.27 (11)	C20—N3—C19—C5	179.93 (9)
C2—C1—C7—N1	153.59 (10)	C6—C5—C19—O3	−26.11 (13)
N1—C7—C1—C6	−28.16 (15)	C4—C5—C19—O3	151.33 (10)
C12—N4—C8—C9	−0.62 (18)	C6—C5—C19—N3	154.80 (9)
C12—N4—C8—N1	−179.82 (11)	N3—C19—C5—C4	−27.77 (13)
C7—N1—C8—N4	−173.33 (11)	C24—N6—C20—C21	−1.56 (15)
C7—N1—C8—C9	7.5 (2)	C24—N6—C20—N3	−179.45 (9)
N4—C8—C9—C10	0.56 (19)	C19—N3—C20—N6	−148.25 (10)
N1—C8—C9—C10	179.66 (11)	C19—N3—C20—C21	33.81 (15)
C8—C9—C10—C11	−0.2 (2)	N6—C20—C21—C22	1.87 (16)
C9—C10—C11—C12	−0.1 (2)	N3—C20—C21—C22	179.58 (10)
C8—N4—C12—C11	0.30 (19)	C20—C21—C22—C23	−0.70 (17)
C10—C11—C12—N4	0.1 (2)	C21—C22—C23—C24	−0.59 (18)
C14—N2—C13—O2	−3.35 (16)	C20—N6—C24—C23	0.12 (16)
C14—N2—C13—C3	175.81 (9)	C22—C23—C24—N6	0.93 (18)

### Hydrogen-bond geometry (Å, º)

**Table:**

*D*—H···*A*	*D*—H	H···*A*	*D*···*A*	*D*—H···*A*
N2—H2···O3^i^	0.898 (14)	2.108 (14)	2.9866 (11)	165.6 (13)
N3—H3···O2^ii^	0.857 (14)	2.054 (14)	2.8781 (11)	160.9 (13)

Symmetry codes: (i) −*x*, −*y*+1, −*z*+1; (ii) −*x*+1, −*y*+1, −*z*+1.

## References

[bb1] Bernstein, J., Davis, R. E., Shimoni, L. & Chang, N.-L. (1995). *Angew. Chem. Int. Ed. Engl.* **34**, 1555–1573.

[bb2] Dolomanov, O. V., Bourhis, L. J., Gildea, R. J., Howard, J. A. K. & Puschmann, H. (2009). *J. Appl. Cryst.* **42**, 339–341.

[bb3] Groom, C. R., Bruno, I. J., Lightfoot, M. P. & Ward, S. C. (2016). *Acta Cryst.* B**72**, 171–179. 10.1107/S2052520616003954 PMC4822653 27048719

[bb4] Jia, T., Zhao, Y., Xing, F., Shao, M., Zhu, S. & Li, M. (2009). *J. Mol. Struct.* **920**, 18–22.

[bb5] Kumar, D. K., Jose, D. A., Dastidar, P. & Das, A. (2004). *Chem. Mater.* **16**, 2332–2335.

[bb6] Luo, X.-Z., Jia, X.-J., Deng, J.-H., Zhong, J.-L., Liu, H.-J., Wang, K.-J. & Zhong, D.-C. (2013). *J. Am. Chem. Soc.* **135**, 11684–11687. 10.1021/ja403002m 23885835

[bb19] Macrae, C. F., Sovago, I., Cottrell, S. J., Galek, P. T. A., McCabe, P., Pidcock, E., Platings, M., Shields, G. P., Stevens, J. S., Towler, M. & Wood, P. A. (2020). *J. Appl. Cryst* **53**, 226–235. 10.1107/S1600576719014092 PMC6998782 32047413

[bb7] Palmans, A. R. A., Vekemans, J. A. J. M., Meijer, E. W., Palmans, A. R. A., Kooijman, H. & Spek, A. L. (1997). *Chem. Commun.* pp. 2247–2248.

[bb8] Rajput, L. & Biradha, K. (2008). *J. Mol. Struct.* **876**, 339–343.

[bb9] Rajput, L. & Biradha, K. (2011). *J. Mol. Struct.* **991**, 97–102.

[bb10] Rigaku OD (2017). *CrysAlis PRO* software system. Rigaku Corporation, Wroclaw, Poland.

[bb11] Senior, L. & Linden, A. (2020*a*). *Polyhedron*, https://doi.org/10.1016/j.poly.2020.114564.

[bb12] Senior, L. & Linden, A. (2020*b*). *Acta Cryst.* C**76**, 562–571. 10.1107/S2053229620006130 32499453

[bb13] Sheldrick, G. M. (2015*a*). *Acta Cryst.* C**71**, 3–8.

[bb14] Sheldrick, G. M. (2015*b*). *Acta Cryst.* A**71**, 3–8.

[bb15] Spek, A. L. (2015). *Acta Cryst.* C**71**, 9–18. 10.1107/S2053229614024929 25567569

[bb16] Spek, A. L. (2020). *Acta Cryst.* E**76**, 1–11. 10.1107/S2056989019016244 PMC6944088 31921444

[bb17] Zhang, L., Dang, L., Luo, F. & Feng, X. (2016). *J. Mol. Struct.* **1106**, 114–120.

[bb18] Zhang, Y., Wang, Q., Xiao, Y.-J., Han, J. & Zhao, X.-L. (2012). *Polyhedron*, **33**, 127–136.

